# Molecular Diagnostic Tools Applied for Assessing Microbial Water Quality

**DOI:** 10.3390/ijerph19095128

**Published:** 2022-04-22

**Authors:** Lisa Paruch

**Affiliations:** Division of Environment and Natural Resources, Norwegian Institute of Bioeconomy Research—NIBIO Oluf Thesens vei 43, 1433 Aas, Norway; lisa.paruch@nibio.no

**Keywords:** microbial water quality, waterborne pathogens, antibiotic-resistance genes (ARGs), end-point polymerase chain reaction (PCR), real-time quantitative PCR (qPCR), DNA microarray, multiplex qPCR (mqPCR), digital droplet PCR (ddPCR), loop-mediated isothermal amplification (LAMP), high-throughput next-generation DNA sequencing (HT-NGS)

## Abstract

Microbial water quality is of vital importance for human, animal, and environmental health. Notably, pathogenically contaminated water can result in serious health problems, such as waterborne outbreaks, which have caused huge economic and social losses. In this context, the prompt detection of microbial contamination becomes essential to enable early warning and timely reaction with proper interventions. Recently, molecular diagnostics have been increasingly employed for the rapid and robust assessment of microbial water quality implicated by various microbial pollutants, e.g., waterborne pathogens and antibiotic-resistance genes (ARGs), imposing the most critical health threats to humans and the environment. Continuous technological advances have led to constant improvements and expansions of molecular methods, such as conventional end-point PCR, DNA microarray, real-time quantitative PCR (qPCR), multiplex qPCR (mqPCR), loop-mediated isothermal amplification (LAMP), digital droplet PCR (ddPCR), and high-throughput next-generation DNA sequencing (HT-NGS). These state-of-the-art molecular approaches largely facilitate the surveillance of microbial water quality in diverse aquatic systems and wastewater. This review provides an up-to-date overview of the advancement of the key molecular tools frequently employed for microbial water quality assessment, with future perspectives on their applications.

## 1. Introduction

Water quality is an essential criterion for all living beings. The provision of clean water remains highly demanding globally, and it constitutes a specific primary goal (No 6 “Clean Water and Sanitation”) among the 17 United Nations Sustainability Development Goals. Quality-compromised water poses serious health risks to humans and animals, either directly via waterborne diseases and/or indirectly via foodborne disease from the contamination of food by, e.g., pathogens in reclaimed water used for irrigation/food production.

Water contaminated with microbial pathogens constitutes a major public threat worldwide, causing over 800,000 deaths each year [1]. The associated global economic loss has been estimated as nearly USD 12 billion per year [2]. Pathogen contaminations have been registered/reported in different types of water, such as freshwater [3,4,5], marine water [6,7,8], drinking water [9,10], reclaimed water [11,12], and wastewater [13,14]. This issue has led to large-scale waterborne epidemic outbreaks in a number of countries worldwide, mainly caused by pathogenic bacteria [9,15,16], viruses [17,18,19], and protozoa [20,21,22].

In recent years, antimicrobial resistance (AMR) has emerged as a major public health threat worldwide. It has been hypothesized that by 2050, this global burden will have caused approximately 10 million deaths. Aquatic systems are identified as the primary reservoirs and hubs for the propagation and transmission of antibiotic-resistant bacteria (ARB) and antibiotic resistance genes (ARGs) [23]. Pathogenic bacteria are more prone to the development of AR due to constant selective pressure and extended dissemination. Antibiotic residues can enter aquatic environments via, e.g., the discharge of wastewater treatment plants (WWTPs) and agricultural runoff (containing livestock manure and organic fertilizers). Both ARB and ARGs have been detected in different water sources, such as groundwater [24,25], surface water [26,27,28], and drinking-water treatment plants and distribution systems [29,30,31]. The ARB (mostly pathogenic) and ARGs (both intracellular and cell-free) present in water can ultimately infect humans and animals through contaminated food, resulting in the reduced efficacy of antibiotics in the treatment of infectious diseases. Thereby, regular monitoring and rapid detection are crucial to ensure water safety.

To facilitate a timely response with effective and appropriate remediation measures, robust and swift identification methods for microbial contaminants in water are crucial, especially upon the onset of an epidemic outbreak. Molecular examinations involve the detection of specific DNA/RNA sequences in target pathogens without culturing. Thus, not only can the result turnaround time be largely shortened, but the assay’s sensitivity, specificity, and reproducibility can also be considerably enhanced compared to traditional culture-dependent microbiological methods, particularly regarding viable but not culturable (VBNC) microbes. With continued technological development, molecular methods have been constantly improved and evolved, from conventional end-point polymerase chain reaction (PCR) to real-time quantitative PCR (qPCR), DNA microarray, digital droplet PCR (ddPCR), loop-mediated isothermal amplification (LAMP), and high-throughput next-generation DNA sequencing (HT-NGS)-based metagenomic approaches. Such technological achievements have contributed to expanding the analysis toolkit used for resolving varied research objectives, such as microbial pollutants in waterbodies or microbial community shifts under anthropogenic impacts and climate change. Rapid and highly sensitive molecular diagnostics offer pivotal information needed for the quality control loop: monitoring–evaluation–action. In this review, the most frequently applied molecular methods for assessing water microbiology are addressed and discussed based on their intrinsic strengths and limitations.

## 2. Presence/Absence Examination by End-Point PCR

**Figure 1:**
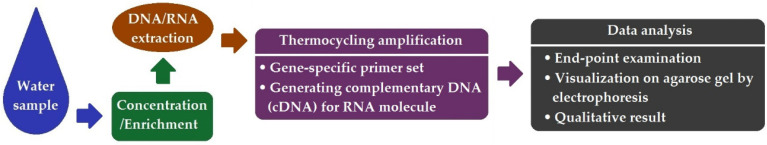
Schematic workflow of conventional PCR.

Conventional end-point PCR can answer the question of the presence/absence of target pathogens in water. It has been broadly used since the 1990s for environmental surveys. Generally, PCR involves three cycling steps, namely, denaturation, annealing, and elongation, to multiply and amplify a specific target DNA sequence. After dozens of amplifying cycles, the target sequence can be exponentially enriched, and the yielded product can be detected and visualized through agarose gel electrophoresis. A schematic workflow is depicted in Figure 1.

Conventional PCR has been used to detect various waterborne pathogens, such as *Escherichia coli* (*E. coli*), *Shigella*, enterotoxigenic *E. coli* (ETEC), *E. coli* O157:H7, *Clostridium perfringens* (*C. perfringens*), *Legionella pneumophila* (*L. pneumophila*), *Campylobacter jejuni* (*C. jejuni*), and *Campylobacter coli* (*C. coli*) [32,33,34,35,36,37]. By targeting a 130 bp DNA region, Pollard et al. [38] detected *E. coli* O157:H7 in water samples, whereas others ran PCR-amplifying fragments of toxin genes *stx1* [39], *stx2* [40], and *eae* [41]. A 166 bp DNA sequence encoding β-d-glucuronidase of the *E. coli uidA* gene was used to characterize various *E. coli* types, e.g., enteroinvasive *E. coli* (EIEC), enteropathogenic *E. coli* (EPEC), and ETEC [42]. The same authors also detected *Vibrio cholerae* (*V. cholerae*) in drinking water sources by PCR targeting the *epsM* gene. Species-specific PCR detected pathogenic toxin-carrying *C. perfringens* in freshwater samples [35]. In another study, a 264 bp fragment was used as the target for a PCR assay to identify six strains of *L. pneumophila* in water [36]. A semi-nested PCR was designed to specifically detect *C. jejuni* and *C. coli* in drinking water and environmental water by targeting intergenic sequences of *flaA* and *flaB* flagellin genes [37]. Xie and colleagues [43] achieved the simultaneous detection of waterborne pathogens *Salmonella* spp., *Pseudomonas aeruginosa (P. aeruginosa)*, *E. coli* O157:H7, and *Bacillus cereus* (*B. cereus*) in environmental water, using developed specific primers for genes *invA*, *ecfX*, *cesB*, and *fliC*. The characterization of pathogenic types of *E. coli* prevalent in river water in Japan was determined by multiplexed PCR and sequencing by examining 14 virulence genes [44]. Additionally, a PCR assay was used for protozoa monitoring, e.g., *Giardia*, Toxoplasma, and *Cryptosporidium*, in raw and drinkable water [45]. Reverse transcription-nested PCR was used to detect human hepatitis virus A and E (HAV and HEV) in groundwater [46]. Additionally, conventional PCR was applied to analyze human adenovirus (HAdV), human enterovirus (hEV), and genogroup A rotavirus (GARV) in Brazilian school tap water samples [47]. Furthermore, ARGs and integron genes were screened in a Chinese aquaculture pond, and an end-point PCR assay revealed high percentages of *blaTEM*, *tetC*, *sulI*, *aadA*, *floR*, and *qnrB*, as well as the integron I gene [48].

Conventional PCR as a culture-independent method is more sensitive and time- and labor-effective compared to traditional culture-based microbial assay. However, only qualitative outcomes (yes/no) can be expected from the assay, as the detection is only performed in the plateau phase. The contamination of PCR products may occur during post-PCR processing, such as agarose gel electrophoresis. Moreover, non-specific products can also be by-generated during amplification, which may complicate the interpretation of the result.

## 3. DNA Microarray

**Figure 2:**
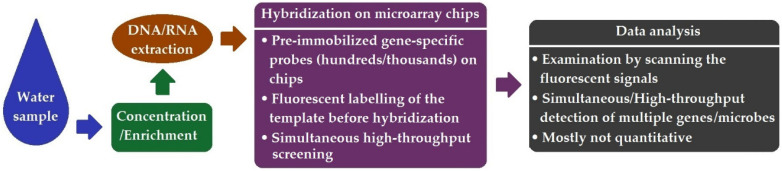
Schematic workflow of DNA microarray.

The microarray, also known as DNA chip or lab-on-chip, was developed in the early- to mid-1990s. DNA microarrays are gene chips containing a high density (over thousands) of nucleic acid probes (genomic DNA, cDNA, or oligonucleotides) in an ordered two-dimensional matrix, which enables the simultaneous detection of multiple target genes in a single assay [49]. Based on hybridization between the immobilized specific probe and the target gene carrying the complementary sequence, the presence/absence of the microorganism of interest can be interrogated by examining the generated fluorescent signal. A schematic diagram presented in Figure 2 indicates the workflow of this method.

DNA microarray was used as a screening tool to assess the occurrence of 941 bacterial pathogens in groundwater [50]. A multigene target microarray was developed to assess the prevalence of waterborne pathogens in a fecally contaminated watershed [51]. Pathogens, including five viruses, nine bacteria, and three eukaryotes, were found to be prevalent in the impaired streams. Gomes et al. [52] developed a DNA microarray carrying 16 implanted probes targeting *P. aeruginosa*, *Staphylococcus aureus* (*S. aureus*), *C. perfringens*, *E. coli*, total and fecal coliforms, and enterococci for water microbiological surveillance. To examine the common waterborne protozoan pathogens, a 21 probe-based microarray was fabricated and successfully detected the three protozoa *Acanthamoeba castellanii* (*A. castellanii*), *Cryptosporidium parvum* (*C. parvum*), and *Giardia intestinalis* (*G. intestinalis*) [53]. Similarly, another assay was developed by Brinkman et al. [54] to simultaneously detect *C. parvum*, *Cryptosporidium hominis* (*C. hominis*), *Enterococcus faecium* (*E. faecium*), *Bacillus anthracis* (*B. anthracis*), and *Francisella tularensis* (*F. tularensis*) in water. Coupled with PCR, the multi-target microarrays containing 780 unique probes targeting 27 DNA and RNA viruses were used to screen and characterize human enteric viruses in community wastewaters [55]. Moreover, a microarray integrated with a panel of virulence genes and antibiotic and heavy-metal-resistance genes was applied to assess the potential risk associated with an impaired agricultural watershed creek [56]. Antibiotic-resistant Enterobacteriaceae were revealed using an AMR-gene-based microarray in fecally contaminated surface urban water, indicating a poor wastewater treatment effect [57].

DNA microarray enables the simultaneous detection of multiple target sequences, displaying a high degree of parallelism (capacity of probes included in a run) and high scalability. However, care must be taken upon the choice of the method due to certain technical limitations, such as high background noise derived from non-specific cross-binding, which can lead to reduced specificity. Additionally, due to the lack of pathogen sequence information, probe design can be challenging. Other potential limitations, such as high costs for a single experiment; limited detection dynamic range; and relatively low sensitivity, accuracy, and reliability compared to qPCR [58,59], also need to be considered. Thus, it is challenging to apply microarrays for the direct examination of waterborne pathogens, which usually present at very low/“dilute” concentrations. To address this challenge, a prior culturing of bacterial pathogens and increasing the volume of the water sample 10 times were proposed by Gomes et al. [52], and these technical modifications led to increased bacterial detection. Additionally, pre-PCR amplification was suggested prior to the microarray to enrich target genes for ease of detection [60,61].

## 4. Quantitative Real-Time PCR (qPCR)

**Figure 3:**
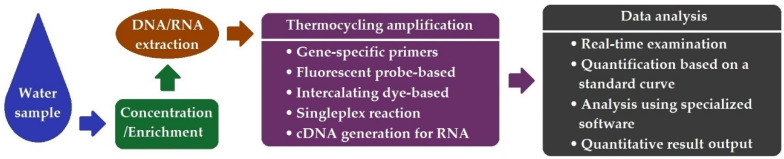
Schematic workflow of qPCR.

The advent of real-time PCR in the late 1990s revolutionized the molecular diagnostic realm. Improved assay sensitivity, accuracy, and specificity, particularly the possibility of rendering quantification, has attracted extensive interest and popularity in use. By recording the fluorescent signal intensity at each amplification cycle in real-time, the concentration of the original target can be determined using a standard curve derived from serial dilutions of gene constructs with pre-known concentrations. Figure 3 illustrates the principal workflow of qPCR.

Both SYBR green- and TaqMan probe-based qPCR systems have been extensively used in water quality examinations for the quantification of multiple waterborne pathogens, including bacteria, viruses, and parasitic protozoa. *Salmonella* spp., *L. pneumophila*, and *P. aeruginosa* were found in high abundances in river water (Jiulong River) using SYBR green-based qPCR [62]. A qPCR assay following a pre-enrichment step detected *Salmonella* spp. and *E. coli* O157:H7 with high sensitivity in surface water [63]. Potable and surface water were found to be contaminated by pathogenic *Enterobacter* in an Indian city (Lucknow) using SYBR green qPCR [64]. The TaqMan qPCR-based assays identified waterborne pathogens, i.e., *C. jejuni*, *C. perfringens*, *Enterococcus faecalis* (*E. faecalis*), and Shiga toxin-producing *E. coli* STEC, in a Norwegian Campylobacter drinking water outbreak in 2019 [15]. Genotypic real-time PCRs were developed to target the small-subunit (SSU) rRNA gene and the 90 kDa heat shock protein (hsp90)-encoding gene for the identification of *Cryptosporidium* spp. in different settings [65]. For a study on waterborne viruses, human enteric viruses, i.e., enterovirus, norovirus, adenovirus, and hepatitis A and E viruses were examined and monitored using different molecular methods, including qPCR, over 1 year in downstream rivers of a WWTP [66]. Real-time PCR has also been widely implemented for AMR examinations in water. Among 23 investigated ARGs, *sul1*, *tetA*, *tetC*, *tetZ*, *gyrA*, *ermF*, *cmlA*, and *blaTEM* were predominant in river waters (Ba River) by SYBR green qPCR [67], associated with persistent antibiotic pollution derived from anthropogenic activities. Another related study set out to search for the link between the sewage source and the affected waters in terms of ARG prevalence in the Brisbane River system [68]. The researchers identified significant correlations between the fecal indicator *E. coli*, target ARGs, and class I integron-integrase (*intI1*) and sewage marker genes, which revealed a direct influence of untreated sewage on ARG occurrence and distribution in downstream water.

It should be noted that for the quantitative microbial source tracking (QMST) of fecal pollution in water, host-specific genetic markers are constantly being developed to determine the fecal origin/s using qPCR [69,70,71,72]. qPCR, as a powerful molecular tool, enables the quantitative examination of the target of interest and presents a higher sensitivity and specificity than conventional PCR. Additionally, less hands-on time (no post-electrophoresis) and high-throughput data analysis capability are additional reasons for the broad use of this approach in diverse research applications. Later, to address the need for differentiation between viable and non-viable microorganisms, viability PCR (v-PCR) was developed. By the simple pre-treatment of the sample using specific intercalating photoreactive chemicals/dyes, such as propidium monoazide (PMA) and ethidium monoazide (EMA), DNA from live cells only can be detected by PCR [73]. For instance, real-time PCR coupled with EMA treatment could selectively monitor viable target bacteria in an aquatic environment [74]. In one study, PMA-qPCR was applied to reliably measure the viable bacterial enteric pathogens, e.g., *E. coli* O157:H7, thermophilic *Campylobacter*, and *Salmonella enterica* (*S. enterica*), in a Canadian river used as drinking water source [75]. Real-time PCR empowers rapid and quantitative target examination with high sensitivity and specificity. For instance, to aid community-level COVID-19 monitoring, wastewater-based epidemiology (WBE) has extensively utilized reverse-transcription qPCR (RT-qPCR) to detect diverse variants of SARS-CoV-2 in wastewater [76]. This WBE-based approach can provide an early warning of the new trend of viral transmission, and support informed pandemic management.

In future, qPCR will continually represent one of the gold-standard molecular methods. In particular, the high costs for equipment, analysis software and consumables (e.g., fluorescent probes) will be drastically reduced, and further technological improvements (e.g., enhanced resistance to PCR inhibitors and minimized background noise signal) will be realized.

## 5. Multiplex qPCR (mqPCR)

**Figure 4:**
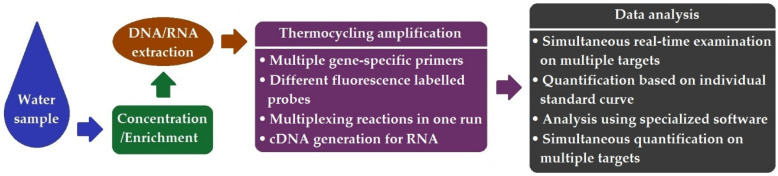
Schematic workflow of mqPCR.

Multiplex qPCR enables multiple gene targets to be detected and quantified simultaneously, representing a much faster and efficient method than singleplex qPCR. To achieve successful mqPCR, primer design is the key factor, since primers must be highly specific and optimized to accommodate the simultaneous detection of various targets in a single reaction. Currently, there are many available molecular software online tools, such as MPprimer [77] and Ultiplex [78], which can be used for primer design to obtain the best mqPCR performance. A technical workflow with specific methodological features of mqPCR is shown in Figure 4.

Multiplex qPCR was developed to identify three species of health-critical waterborne pathogen *Campylobacter* (i.e., *C. jejuni*, *C. coli*, and *C. lari*) concomitantly in food and water [79]. In combination with PMA treatment, a multiplex qPCR assay managed to detect viable *L. pneumophila*, *S. typhimurium*, and *S. aureus* in environmental water [80]. Another mqPCR was developed to concurrently detect protozoan pathogens *Giardia lamblia* (*G. lamblia*) and *C. parvum* in water samples [81], with detection limits of one cyst of *G. lamblia* and a single oocyst of *C. parvum*. Kang et al. [82] compared mqPCR with respective individual qPCR on waterborne viruses (norovirus genogroup I, norovirus genogroup II, rotavirus group A, hepatitis A virus, and coxsackievirus group B1,) and reported a comparable sensitivity and specificity for the two approaches. A multiplex RT-qPCR assay was developed to detect SARS-CoV-2 in wastewater by targeting various genes, i.e., *N1, N3* and *S* genes [83]. Following a simple viral capture and RNA extraction, multiplex RT-qPCR was used to detect SARS-CoV-2 *N1, N2* and *E* genes in wastewater and provided first-hand data for WBE monitoring of COVID-19 spread in communities [84].

Multiplex qPCR has also been used for AMR studies, e.g., assessing the impacts of hospital wastewater on receiving communal sewage system in terms of ARG concentration changes [85]. From this study, advanced wastewater treatment technology, such as membrane bioreactor treatment (MBR), was identified as an efficient method to mitigate ARGs in wastewater. In addition, a study on ARG profiling across the Rhine River within three European countries was conducted using a developed mqPCR by targeting 13 ARGs and *intI1*, which disclosed the tempo-spatial variabilities [86].

As revealed by the studies mentioned above, mqPCR substantially improves the assay throughput by combining multiple qPCR reactions in one run. In this way, less input material is used, which is practically important for some cases when only limited molecular material is available for assay. Apparently, the time and cost efficiencies of mqPCR can be remarkably improved through the multiplexing approach. However, certain cautions need to be taken when adopting this method. For instance, due to the high potential of primer interactions, the requirements for primer specificity and the compatibility of all primer sets are decisively high to ensure a successful assay. Additionally, there are choice limitations on the types and numbers of targets, of which not all are compatible to be included in the same run. Thus, stringent assay design and adequate optimization are crucial to attain a successful mqPCR before it can be confidently applied in environmental surveys.

## 6. Loop-Mediated Isothermal Amplification (LAMP)

**Figure 5:**
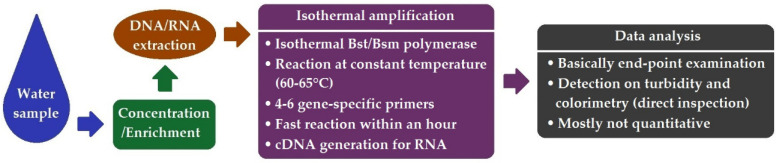
Schematic workflow of LAMP.

LAMP is an isothermal amplification method which was first introduced in 2000 [87]. Different from the PCR technology, which amplifies a target at varied temperatures in a thermocycling mode, LAMP amplifies the target sequence at a constant temperature of 60–65 °C. Relying on strong strand-displacement DNA polymerase (e.g., Bsm polymerase) and four or six specifically designed primers, six or eight regions of a given target gene can be recognized and amplified towards an augmented analytical specificity. The amplification signals can be detected by the photometry of the reaction turbidity or by the colorimetry of the fluorescent intensity of intercalating dyes [88]. This approach offers a rapid (within 1 h), low-cost, easier to use and thermocycler-free alternative method for PCR. Figure 5 presents the schematic workflow of LAMP.

LAMP-based assays have been widely applied for the clinical pathogen diagnostics of infectious diseases and show comparable sensitivity and specificity to PCR [89,90,91,92]. It is noteworthy that reverse-transcription LAMP (RT-LAMP) has been developed and widely implemented as one of the standard diagnostic methods for the rapid detection of SARS-CoV-2 in human saliva [93,94,95]. Moreover, LAMP has also been devised to detect water- and food-borne pathogens, e.g., *Salmonella* spp. [96], *C. jejuni* and *C. coli* [97], as well as *C. perfringens* [98]. By the simple visualization of the LAMP product, *V. cholerae* can be easily detected in water samples [99]. Similarly, VBNC *V. cholerae*, in low numbers in environmental waters, can be assessed using LAMP, which is more sensitive and rapid than the PCR method [100]. Notably, RT-LAMP has been used as a simple and fast alternative method to detect SARS-CoV-2 in wastewater globally [101,102,103,104], which greatly supported local WBE-based pandemic surveillance. Interestingly, LAMP combined with a hydroxynaphtol blue (HBN)-based detection method was used as an on-site approach to detect *L. pneumophila* and other *Legionella* spp. [105]. Due to its rapid, simple setup and portable features, LAMP can be used for convenient field-deployable systems; for instance, a most probable number (MPN)-based LAMP system was applied to quantify waterborne *E. coli* and *E. faecalis* bacteria without DNA extraction [106]. Furthermore, LAMP was adopted for the development of biosensors, offering the on-site and direct detection of pathogens in environmental samples [107]. In another study, LAMP-based microchips integrated with a charge-coupled device (CCD) fluorescence imaging system were developed to rapidly detect waterborne pathogens at a low cost [108]. More recently, Jin et al. [109] devised a LAMP-based microfluidic chip (on-chip LAMP) to simultaneously detect 10 waterborne pathogens. Interestingly, a portable smartphone-based particle diffusometry (PD) platform was developed to detect LAMP-enriched *V. cholerae* in waters, with high (over 90%) sensitivity, specificity, and accuracy [110].

From the application perspective, LAMP has great potential/benefit for the on-site biosensing and biomonitoring of environmental pathogens on point-of-survey or in resource-limiting regions. This is mainly due to the fact that LAMP is a rapid, sensitive, and low-cost method. The assay is convenient to setup without the need for a thermocycler; thus, it is suitable for field applications. Nevertheless, certain cautions need to be taken regarding its technical aspects, e.g., it is rather challenging to make a perfect design of the primer sets to accommodate all the requirements and restrictions relating to, e.g., the choice of the target site, the limited size of the amplified genetic region, or the non-specific amplifications [107], as there is a larger chance to obtain primer dimers and hairpins, leading to false-positive results [111]. Additionally, cross-contamination may occur upon end-point analysis with post-amplification processes [112]. Moreover, the final results judged by the visual observation of turbidity and color changes tend to be more subjective than objective.

## 7. Droplet Digital PCR (ddPCR)

**Figure 6:**
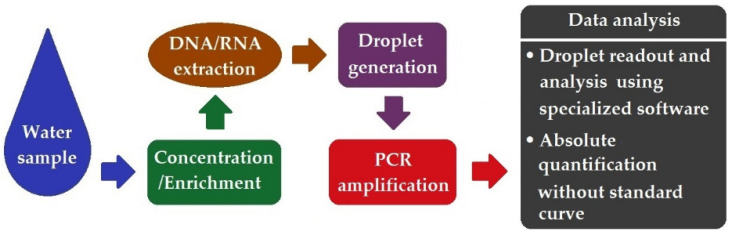
Schematic workflow of ddPCR.

More recently, a novel PCR-based technology, i.e., droplet digital PCR (ddPCR), has appeared, which allows for the absolute quantification of DNA without the need for standard genetic references. As the third generation of PCR technology, ddPCR relies on the massive partitioning of a DNA template into thousands of water–oil emulsion droplets before proceeding to PCR amplification. The final signal readout is carried out at the endpoint of amplification. A schematic workflow of ddPCR is illustrated in Figure 6.

Droplet digital PCR provides more sensitive, precise, and reproducible data than qPCR and exhibits more tolerance to PCR inhibitory substances as usually present in environmental samples [113]. This technology can thus be used for the quantification of a target at an extremely low concentration, e.g., SARS-CoV-2 in wastewater [114,115]. Since it has become commercially available in 2011, it has been widely used in clinical applications and environmental research [116,117,118]. Zoonotic bacterial pathogens, such as *Salmonella* spp., *C. jejuni*, and *Listeria monocytogenes* (*L. monocytogenes*), were quantified by ddPCR in water samples [119]. Salmonella at low concentrations in river sediments was examined by ddPCR, with improved higher sensitivity compared to qPCR [120]. Moreover, a ddPCR assay was developed to simultaneously detect fish pathogens, i.e., *Flavobacterium psychrophilum* (*F. psychrophilum*) and *Yersinia ruckeri* (*Y. ruckeri*), in aquaculture recirculated water [121]. Another common waterborne pathogen, *L. pneumophila*, was effectively detected in water samples, indicating a higher accuracy than qPCR [122]. Regarding waterborne viruses, their detection and quantification are highly challenging since their abundances are usually low in water. Rotavirus, an RNA virus, could be quantified using one-step reverse-transcription ddPCR (RT-ddPCR) in various types of water samples [123]. For the surveillance of the currently prevalent SARS-CoV-2 viruses in wastewater and to support WBE studies, several RT-ddPCR detection methods have been developed to timely and accurately enumerate both wildtype and variants of concerns by targeting varied viral genes. RT-ddPCR was used to detect the mutation of SARS-CoV-2 N501Y in the community, and its applicability for monitoring of new emerging variants was indicated [114]. By targeting *E* and *ORF1ab* genes, optimum primer and probe systems were selected and used for the ddPCR determination of SARS-CoV-2 concentration in raw sewage [124]. Moreover, RT-ddPCR shows higher sensitivity and specificity than RT-qPCR in the quantification of SARS-CoV-2, with an improved detection limit and accuracy [115,125]. In terms of ARG monitoring, Wang et al. [126] implemented ddPCR to identify six clinically relevant ARGs and *intI1* in river water, of which *blaTEM*, *strB*, *aadA*, and *intI1* were detected at high abundances. Similarly, high levels of *blaCTX-M-1*, *ermB*, *sul1*, *tetB*, *tetM*, *tetX*, and *intI1* were found in irrigation return flows with a direct connection to surface waters [127].

The ddPCR empowers molecular diagnostic studies with unprecedented advantages, and undoubtably, it outperforms traditional qPCR with improved sensitivity, precision, and reproducibility, especially for targets at extremely low concentrations, such as SARS-CoV-2 in wastewater. Additionally, it is less prone to PCR inhibitors, and delivers absolute quantification without a standard curve. In the future, this technology can be more widely adopted and exploited for environmental studies, making it a routine test just like qPCR, once the costs for the entire operating system and chemical reagents have been significantly reduced (affordable for more end-users) and the sample’s handling capacity/assay throughput and dynamic range have been profoundly upgraded.

## 8. Next-Generation Sequencing (NGS)-Based Method

**Figure 7:**
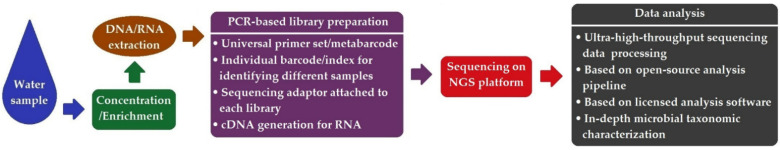
Schematic workflow of NGS.

The advent of next-generation sequencing signifies a technology revolution manifesting the assay platform transition from the first-generation Sanger sequencing to the second generation, reaching ultra-high throughput levels. NGS encompasses an array of DNA and RNA sequencing technologies that provide massively parallel sequencing with extra-high taxonomic resolution. Figure 7 illustrates the principal workflow of NGS methods.

The NGS-based multi-omics studies cover different method categories, i.e., whole-genome sequencing (WGS), metagenomic sequencing, meta-transcriptomic sequencing, and targeted amplicon sequencing. Sequencing operation systems, such as Illumina (MiSeq and HiSeq), 454 pyrosequencing, Ion Torrent, Nanopore and PacBio, represent the most widely employed technical platforms at present [128]. Leveraging NGS technologies, the complex environmental microbial populations together with their community structures and functional capacities can be deeply explored and dissected, making them new powerful tools to detect and map microbial pathogens in wastewater and WWTPs [129,130,131], fishponds [132], sea water [133], spring water [134], cooling tower water [135], recreational water [136,137,138], freshwater [139], and irrigation water [140,141]. Recently, the simultaneous detection of 14 water pathogen target genes was conducted using developed multiplex PCR-coupled amplicon sequencing [142]. Metabarcoding by targeting 18S rRNA was applied for the detection of protozoan pathogens, e.g., *Cryptosporidium* in WWTPs in Australia [130] and *Cryptosporidium* spp., *Giardia* spp., and *Toxoplasma gondii* (*T. gondii*) in shellfish [132]. Deep amplicon sequencing by targeting the 16S rRNA gene was primarily used for bacterial pathogen detection, such as for the highly abundant *Acinetobacter johnsonii* (*A. johnsonii*), *Mycobacterium avium* (*M. avium*), and *Aeromonas* spp. in urban recreational water [137], for *Legionella* profiling in freshwater [139], and *Aeromonas* and *Salmonella* in irrigation water [141]. Metagenomic analysis leveraging the Illumina MiSeq platform was applied to characterize pathogenic viruses, bacteria, and protozoa in irrigation water [140] and to identify high quantities of *Mycobacterium* and hepatitis A and E in the effluent of a sewage treatment plant [131]. It is noteworthy that NGS-based WBE has successfully facilitated the monitoring of the spread and extent of genetic diversity of SARS-CoV-2 variants in wastewater worldwide [143,144,145,146,147]. It is a proven effective tool for verifying the RT-qPCR examination of SARS-CoV-2, and provides in-depth viral mutation mapping through whole-genome sequencing. Moreover, the prevalence of antibiotic-resistant pathogens (pathogens carrying ARGs) in aquatic environments was revealed by metagenomics using Illumina and Oxford Nanopore systems [148,149,150].

In addition to pathogen detection, NGS technologies have helped to dissect the aquatic microbial community composition as a proxy for microbial water quality assessment in various water environments, e.g., rivers and sediments, drinking water distribution systems, fish farming ponds, lakes, recreational water, rainwater, natural wetlands, marine, estuaries, coastal waters, reservoirs, lagoons, and hot springs, under varied exogenous impacts (e.g., miscellaneous pollution from fecal contamination, fertilizing and mining, WWTP discharge, landfill leachate, anthropogenic activities, and environmental variabilities) [151,152,153,154,155,156,157,158,159,160,161,162,163,164,165]. Furthermore, the dynamic changes in microbial diversity under a variety of anthropogenic, geographic, seasonal, and climatic impacts can also be assessed using the NGS approach to elucidate the observed spatiotemporal variabilities [156,162,166,167,168,169,170,171].

NGS-based approaches are proven powerful molecular methods for environmental research, rendering ultra-high data throughput and in-depth taxonomic characterization. However, certain technical aspects need to be further addressed for improvements. For instance, DNA/RNA extraction is a crucial step for downstream molecular analysis, which is equally important for all kinds of DNA/RNA-based methods. Various extraction methods for isolating environmental DNA/RNA (eDNA/eRNA) from diverse matrices (e.g., soil and water) have been well-described and summarized in the published review reports [172,173,174]. Among them, the phenol–chloroform extraction method and a wide variety of commercial purification kits have been broadly employed in numerous aquatic ecosystem studies. The choice of the extraction method depends primarily on the water type; thus, the eDNA/eRNA extraction protocols need to be optimized and modified to maximize the final yield. In addition to this, other technical aspects need to be improved, e.g., streamlined sequencing library preparation, reduced sequencing errors derived from PCR biases, and standard and simplified bioinformatic analysis platforms, as well as well-updated and curated reference databases for in-depth taxonomic characterization and functional annotation. The major methodological pros and cons of NGS along with the other aforementioned methods are summarized in Table 1. Encouragingly, the arrival of the game-changing third-generation DNA sequencing (TGS) technologies, e.g., Nanopore and PacBio platforms, enables longer sequence reads to be generated to simplify genome assembly (particularly for pathogen characterization) and runs sequencing by synthesis (genuine single-molecular sequencing) excluding the PCR step (to avoid PCR artefacts). This significant technological advancement will usher in a new era of application development within environmental research.

## 9. The Surveillance of SARS-CoV-2 in Wastewater Using Different Molecular Methods—A Showcase

The global fight against COVID-19 is still ongoing. The unprecedented pandemic is posing the toughest threat to global health and social–economic growth. SARS-CoV-2, an enveloped and single-stranded RNA virus (belonging to the *Betacoronavirus* family), is the etiological agent of COVID-19 [175]. Although it is a respiratory tract infectious virus, it is also present in stool and urine samples [176]. Both viral RNA and viable virus are found in sewage systems [177]. Thus, WBE has been broadly used to monitor the evolution of the virus and facilitate the early detection of new outbreaks. In this regard, different molecular methods have been developed and exploited for the rapid diagnostics of SARS-CoV-2 in wastewater.

Among all the applied molecular methods, RT-qPCR has been mostly used to detect SARS-CoV-2 in wastewater, representing the “gold standard” approach. Most assays have targeted the nucleocapsid genes, e.g., *N1*, *N2*, *N3*, and envelope (*E*) gene [178,179,180], and *ORF1ab* [181,182,183]. Moreover, multiplexed RT-qPCR analyses were also developed to detect *N1*, *N2*, and *E* genes [84], and *N1*, *N3*, and spike (*S*) genes [83] in wastewater. RT-qPCR has greatly facilitated the robust detection of the virus. However, in some cases, due to the extremely low viral load, qPCR was unable to detect the virus (false-negative results). Digital droplet PCR has proven to be an ultrasensitive approach with enhanced detection limit and assay accuracy for the detection of SARS-CoV-2 and monitoring of the mutations in wastewater [114,115,125]. This method exhibited greater overall sensitivity and specificity than traditional RT-PCR in the detection of low-level SARS-CoV-2 and the identification of viral spread trends at an early stage. However, the instrument and consumables are still very expensive, which may not be accessible for research units with limited resources. Notably, RT-LAMP provides a simple, fast, and cheaper alternative to detect the virus in wastewater [101,102,103,104]. The assay normally takes less than an hour, and the results can be easily determined by naked-eye inspection, demonstrating great potential to support a rapid response to any new trends in viral spread. However, due to the need for more primers (4–6) used for amplification, ideal assay design can be very challenging. Additionally, the result readout by visual inspection is prone to subjectivity.

To obtain more in-depth knowledge on the genetic diversity of SARS-CoV-2 during the COVID-19 pandemic, NGS has been used to profile the viral mutations present in wastewater across infected communities [143,144,145,146,147]. The high-throughput NGS approach enables the in-depth surveillance of the pandemic’s evolution, as it is able to identify and track the spread of viral variants at the community level. Meanwhile, it can also be used to verify the RT-qPCR examination of SARS-CoV-2 and thus to mitigate false-positive results [76]. However, to prepare the environmental samples for sequencing, and moreover, to analyze extra-large dataset with millions of sequencing reads, specialized technical skills and sufficient bioinformatics expertise are required [49]. To this end, WBE-based COVID-19 surveillance demonstrates that diverse molecular methods can be utilized for the rapid and early detection of SARS-CoV-2 and the emergence of new variants, and ultimately, support decision-making management with informed pandemic evolvement.

## 10. Conclusions and Future Perspectives

This review highlights the feasibility and versatility of molecular diagnostic tools used for the surveillance of waterborne microorganisms posing a threat to human and environmental health. Ever-evolving molecular technologies are offering an increasing number of technical options for the qualitative and quantitative assessment of the key components related to aquatic microbial quality, e.g., waterborne pathogens, ARGs, and microbial community dynamics under wide-ranging anthropogenic and environmental impacts. As revealed through this literature review, molecular methods have undergone constant development and succession to meet variable application purposes and requirements. Indeed, each method has own technical characteristics and provides distinct detection platform, such as:

- End-point PCR can facilitate the “presence/absence” diagnostics of target species of interests and is superior to traditional culture-based characterization considering time and labor aspects.
- DNA microarray enables the simultaneous detection of multiple targets in a single run, offering a high degree of assay parallelism.
- Both qPCR and mqPCR can provide quantitative and more rapid and sensitive assays than conventional PCR, and are among the most applied methods for water pathogen detection.
- LAMP offers a unique isothermal amplification-based detection approach and has shown great potential for the development of novel affordable and portable biosensors for on-site water quality biomonitoring.
- The emergence of ddPCR symbolized a significant concept revolution and technology leap in molecular diagnostics. It holds great promise in realizing the precise quantification of microbial pathogens sparsely present in water, with ultra-high sensitivity, precision, and reproducibility.
- With the use of NGS-based high-throughput approaches, e.g., amplicon deep sequencing, metagenomics, meta-transcriptomics, and WGS, multiple environmental pathogens can be simultaneously characterized in depth (at the genus or species level) in various waters and wastewater. Moreover, the structure and functioning of a microbial community can be deciphered using HT-NGS, facilitating the assessment of microbial water quality and the identification of the major anthropogenic or environmental drivers responsible for ecosystem fluctuations.

Notably, each method has both strengths and limitations, resulting in trade-offs in the choice of method, which should rely largely on the research questions to be solved. Practically, different methods can be allied and applied synergistically to boost the assay performance; for example, a pre-PCR step can be performed to enrich the target molecules and facilitate the subsequent microarray assay with an elevated detection rate. Both qPCR and ddPCR can be performed in parallel to render data comparison, cross-validation, and result translation, whereas LAMP has been incorporated into DNA microarrays for novel biosensor development. Through NGS data mining, new microbial indicators and genetic markers can be identified for new qPCR assay setups. Apparently, with more robust molecular tools becoming available and undergoing constant improvement, more powerful and easy-to-go assays can be developed in the near future, which will encourage a more frequent and broader implementation of these tools in environmental research. In this context, this review is projected to enlarge the scope of knowledge on the molecular toolkit used for water microbial quality assessment, through furnishing the most up-to-date progress in this field. Leveraged by these advanced methods, novel and profound research findings will be continuously generated. In turn, this will stimulate further methodological improvement, and furthermore, the development of new technologies. Yet, one of the major obstacles/limitations impeding the broad implementation of the methods lies in the remaining high costs of the specialized instrument and chemical reagents. Moreover, proceeding with the molecular analyses requires well-trained personnel with laboratory experience and sufficient bioinformatics skills (e.g., molecular analysis, primer design, and sequencing data processing).

Another important issue is how to, in practice, integrate DNA-based tools into standard water quality monitoring regimes on a routine basis. Undoubtedly, it is beneficial to combine biotic data with abiotic environmental measures for more systematic water quality assessment. In this respect, guidelines for water quality surveillance should be updated and better strengthened, by incorporating microbial quality assessment into their standard protocols. Recruiting microbes as sentinels for regular water quality examination and pollution control/identification (e.g., emerging contaminants) represents a simple, fast, and effective approach, which is largely supported by molecular tools. For instance, RT-qPCR, RT-ddPCR, and NGS have been successfully employed for WBE-based SARS-CoV-2 monitoring to serve community-level pandemic management. Furthermore, by integrating the molecular analysis data into the epidemiological modelling system, the spread of disease can be predicted. Finally, there is an urgent need to standardize the involved technical procedures (e.g., water sample collection and preservation, water concentration/enrichment, DNA/RNA extraction methods, qPCR, and NGS data analysis protocols) to facilitate cross-laboratory, -country, and -continent data comparison and technology transfer.

## Figures and Tables

**Table 1:** Summary of pros and cons of the key molecular diagnostics applied for microbial water quality assessment.

Method	Pros	Cons
End-point PCR	Culture-independent Time- and labor-effective Improved assay sensitivity compared to traditional culture-based method	Non-quantitative, only qualitative (presence/absence) analysis Post-PCR processing, i.e., electrophoresis Non-specific amplifying product
DNA microarray	Simultaneous detection of multiple target genes Increased assay capacity and scalability	High background-level hybridization leading to reduced assay specificity Lack of pathogen sequence information for probe design Limited detection dynamic range Not always quantitative
Quantitative real-time PCR (qPCR)	Rapid detection approach High sensitivity, accuracy, and specificity Quantitative examination	Sensitive to PCR inhibitors High costs of instrument, analysis software and consumables Requirements for special skills and expertise on assay design and data analysis
Multiplex qPCR (mqPCR)	Improved assay throughput by simultaneous detection multiple target sequences Using less input material Enhanced time- and cost-efficiencies	High requirements for assay design Uneven amplification efficiency between different targets Prone to primer interaction and reagent competition
Loop-mediated isothermal amplification (LAMP)	Rapid assay (less than one hour) Isothermal (no need for a thermocycler) Good selectivity and sensitivity Simple assay setup and result readout Low cost Portable for field applications	Challenge to perfectly design primers Non-specific amplification and false-positive results caused by primer dimers and hairpins Prone to cross-contamination upon final readout Subjective due to visual inspection of reaction changes
Droplet digital PCR (ddPCR)	More sensitive, precise, and reproducible than qPCR Superable for detection of target at low abundance High tolerance to PCR inhibitors No need for standard curve	Very high costs for instruments and reagents Low assay throughput Limited dynamic range Complex upon multiplexing
Next-generation sequencing (NGS)-based	Ultra-high throughput In-depth taxonomic characterization Enables entire microbial community profiling, and *α*- and *β*-diversity comparison High accuracy Time and cost effective	Expensive sequencing equipment and high run cost Less standardized and customized sample preparation procedures for different water types Sequencing errors resulting from PCR bias Intricate sequencing data processing Requiring proficient bioinformatics expertise

## Data Availability

Not applicable.
